# Using Group Chats to Drive Behavior Change in Digital Health Interventions: Scoping Review and Realist Synthesis

**DOI:** 10.2196/88911

**Published:** 2026-04-13

**Authors:** Hein Thu, Felicia Jia Hui Chan, Jumana Hashim, Shahmir H Ali

**Affiliations:** 1 Burma Academy, Yangon, Myanmar; 2 Saw Swee Hock School of Public Health, National University of Singapore, Tahir Foundation Building, 12 Science Drive 2, #10-01, Singapore, 117549, Singapore, 65 87918862; 3 Behavioural and Implementation Science Interventions, Yong Loo Lin School of Medicine, National University of Singapore, Singapore, Singapore

**Keywords:** group chats, health behavior, social cognitive theory, realist synthesis, scoping review

## Abstract

**Background:**

Group chats on platforms such as WhatsApp (Meta Platforms, Inc), WeChat (Tencent Holdings Limited), and Telegram (Telegram FZ-LLC) are central to everyday communication in many settings, including across low- and middle-income countries and among groups often overlooked by one-to-one or app-based digital health tools. Yet their roles and underlying mechanisms as intentionally designed health interventions have not been comprehensively examined.

**Objective:**

This scoping review with realist synthesis aimed to map the characteristics and reported outcomes of group chat–based health interventions and contextual conditions shaping their outcomes. It mapped intervention characteristics and identified context-mechanism-outcome (CMO) configurations driving behavior change.

**Methods:**

We included empirical studies in which group chats were the primary digital component of the health intervention. Searches of PubMed, Embase, MEDLINE, Web of Science, and Scopus (2005‐2026) and reference screening identified eligible studies. Data were extracted on study characteristics, health domains, settings, participants, platform, intervention features, and outcome domains. For the realist synthesis, CMO configurations were analyzed using reflexive thematic analysis informed by social cognitive theory and synthesized into a final program theory.

**Results:**

Eighty-one studies were included. Publications increased sharply after 2020, with evidence clustering in mental health (25/81, 30.9%) and maternal and child health (20/81, 24.7%). Most studies enrolled adult participants, including pregnant or postpartum women, caregivers, and people managing chronic conditions. WhatsApp (38/81, 46.9%) and WeChat (21/81, 25.9%) were the most frequently studied platforms. Interventions were commonly delivered in clinical or community settings and typically involved mixed membership groups that included health professionals alongside participants (68/81, 84.0%). The realist synthesis identified 12 recurring CMO configurations across 5 domains: capability and actionability, confidence and motivation, modeling and norms, safe and supportive environment and access, and self-regulation and maintenance. These mechanisms were activated or suppressed by facilitation quality, group composition, cultural alignment, technological access, and social norms. The final program theory depicts group chats as dynamic systems where access, facilitation, and group structure shape how the domains amplify or dampen one another, explaining shifts from low engagement to high trust spaces sustaining behavior change.

**Conclusions:**

This review integrates descriptive mapping of interventions with realist program theory to explain how context and facilitation activate mechanisms of change, unlike prior reviews that have focused narrowly on effectiveness of digital modalities. This dual approach provides a practical, mechanism-focused basis for designing and scaling group chat interventions in practice, particularly given their potential as a low-cost, high-reach strategy embeddable within broader digital health programs. Realizing this potential depends on treating group chats as purposefully designed social environments, with deliberate attention to facilitation quality, equity-oriented access, and alignment between group structure, norms, and communication practices.

## Introduction

The global spread of smartphones, mobile internet, and messaging apps has normalized digital communication and reshaped health promotion and care delivery [1]. Digital tools now support prevention, screening, chronic care, and adherence at scale and low cost [2-4]. Social media expanded interactive engagement, and 5.5 billion people were online in 2024, about 68% of the global population [3,4]. Many reviews still pool disparate modalities such as apps, SMS, and social media under 1 digital health label, which obscures modality-specific mechanisms and user dynamics [5]. Relatedly, artificial intelligence (AI)–enabled conversational agents (chatbots) have also emerged as a rapidly growing approach in digital behavioral interventions, with recent syntheses reporting generally modest, heterogeneous effects across outcomes and highlighting ongoing considerations around safety, privacy, and governance in health contexts [6].

Given this expanding and heterogeneous landscape, a clearer emphasis on mechanisms is needed. Behavior change theories are often invoked to guide digital design and interpretation, for example, observational learning, social reinforcement, and self-efficacy, but many studies reference theory briefly and rarely test mechanisms within digital environments [7-11]. Realist synthesis has grown as a way to explain what works, for whom, in what contexts, and how, advancing context-mechanism-outcome (CMO) explanations for digital interventions [12-14], yet most syntheses remain modality agnostic (ie, do not disaggregate by technology type, such as apps vs SMS vs group chats), focus primarily on evidence from more complex, app-based interventions (which may be less translatable in low- and middle-income country (LMIC) settings where simpler messaging tools predominate), or lack intensive exploration of digital technologies that engage more complex forms of behavior change (ie, those involving social and community processes).

Social media messaging has now become an integral part of the digital health landscape of many countries, particularly LMICs [15], where platforms such as WhatsApp (Meta Platforms, Inc), Telegram (Telegram FZ-LLC), Viber (Rakuten, Inc), Signal (Signal Technology Foundation), and WeChat (Tencent Holdings Limited) are widely used by patients and providers. More than 85% of physicians and nurses own smartphones or tablets, and 60% to 80% of clinical staff use text messaging for patient communication [16]. In LMIC settings, messaging apps have supported health education and COVID-19 response, and about 80% of households have mobile phone access [17,18]. At the same time, much recent innovation in digital behavioral interventions has focused on one-to-one formats, including conversational agents and AI-enabled chatbots that deliver tailored coaching through dyadic dialogue [19]. However, group chats are a qualitatively different intervention environment, as they are multiparty, relationship-embedded spaces where norms, privacy, and trust are coproduced in real time, so design lessons from one-to-one chatbot models may not translate directly.

Within this ecosystem, group chats on WhatsApp, Telegram, WeChat, LINE (LINE Corporation), and Signal are multiuser threads that enable real-time, small-group, conversational interaction. This format differs from one-to-one messaging (the dominant configuration for most chatbot interventions), which is dyadic and linear, and from broadcast social media, which is asymmetric and feed-based. Participants exchange messages, images, and voice notes within semiprivate family, peer, or community networks, which affords familiarity, immediacy, and intimacy [20,21]. In many LMIC settings, group chats (particularly on platforms such as WhatsApp) are a particularly important part of the digital health environment [22]. Importantly, unlike other types of digital platforms, they often remain accessible to older adults and lower literacy users because they are low bandwidth and simple to use [20,23]. Compared with app-centric self-tracking or SMS reminders, group chats foreground interaction over individual logging and mirror offline support structures such as trust, reciprocity, and collective efficacy.

Despite widespread everyday use, research on group chats has largely been observational [24,25], often focusing on understanding social support dynamics or how health information or misinformation spreads in these spaces. What is missing is a consolidated synthesis of group chats as a primary intervention platform. Evidence remains fragmented on where, how, and for whom group chats are used intentionally to deliver health content, coaching, or peer support, and on how facilitation, privacy, culture, and gender norms shape participation. This gap matters because group chats can engage populations that are often excluded by data-heavy or app-centric models and can better reflect the interpersonal and community networks that drive health behavior change. As noted in prior reviews, this is particularly important in LMIC contexts, where group chats embedded within familiar, everyday social messaging platforms may be more feasible and acceptable than standalone health apps, yet empirical evidence on their implementation and the mechanisms through which they operate remains limited and fragmented [26-28]. A focused synthesis is therefore needed to understand which design and delivery elements are transferable across diverse settings or require adaptation depending on contexts [29].

To address these gaps, this scoping review with realist synthesis aims to map and synthesize the existing literature on group chat–based health interventions and to explain how these interventions work in practice. Specifically, the scoping review describes the characteristics of group chat interventions across studies, including platform type, key design features, target populations, health domains, and geographic distribution, and summarizes the outcomes reported across behavioral, psychosocial, and clinical domains. The realist synthesis then explores the mechanisms and contextual conditions that shape engagement and behavior change, including facilitation style, group composition, trust, cultural norms, and platform features. Together, these approaches identify where evidence is concentrated, highlight key gaps, and offer an explanatory framework to inform the design and adaptation of future group chat interventions.

## Methods

### Study Design and Reporting Frameworks

This review combined a scoping review with a realist synthesis to generate both descriptive and explanatory insights into group chat–based health interventions. The scoping review mapped the scope, characteristics, and distribution of the evidence base, including intervention design features, platforms, target populations, health domains, and settings. It also summarized the range of outcomes reported across studies, including behavioral, psychosocial, and clinical outcomes, to clarify what has been evaluated and where evidence remains limited. The realist synthesis complemented this by examining how and why group chat interventions achieved outcomes, drawing on qualitative, mixed methods, and process data to develop and refine program theories. The scoping review component was reported in accordance with the PRISMA-ScR (Preferred Reporting Items for Systematic Reviews and Meta-Analyses extension for Scoping Reviews) [30]; the completed reporting checklist is provided in Checklist 1. The search was reported in accordance with PRISMA-S (Preferred Reporting Items for Systematic Reviews and Meta-Analyses literature search extension) [31]; the full search checklist and search strings are provided in Checklist 2. The realist synthesis was reported in accordance with RAMESES II (Realist And Meta-narrative Evidence Syntheses: Evolving Standards II) standards [32]. The review was prospectively registered in PROSPERO (CRD420251082787) as a systematic review, but it was conducted as a scoping review to better reflect the mapping aims and breadth of the evidence. Quality appraisal was also planned initially using Cochrane RoB-1 and ROBINS-I. However, no formal critical appraisal or risk-of-bias assessment was conducted, as the scoping review aimed to map and characterize the evidence base rather than exclude studies based on study quality.

### Development of Initial Program Theories

The Initial Program Theories (IPTs) outlined hypothesized pathways through which participation in group chats might have influenced health behavior. These IPTs were developed prior to data extraction and served as a starting framework for realist analysis, subsequently refined as evidence was synthesized. IPT development began with a scoping review of literature on digital health, peer support, and messaging-based interventions to identify recurring contexts (eg, facilitation, message cadence, and trust) and mechanisms (eg, modeling, reinforcement, accountability, and belonging). These insights were organized using social cognitive theory (SCT) [33], which was chosen as the guiding framework because it is an established and widely applied model for understanding mechanisms of behavior change in digital health interventions and for informing their design [34]. SCT links social interaction, observational learning, and reinforcement (core processes in group chat environments) to behavior change, providing a strong theoretical basis for explaining how digital communication can shape motivation, confidence, and action. Within this framework, constructs such as self-efficacy, observational learning, behavioral capability, reinforcement, environment, and self-regulation were mapped to the interpersonal and contextual dynamics of group chats. Provisional CMO propositions were then articulated to capture how design features and social processes might generate change; for example, how active facilitation could create accountability, or how peer similarity and trust might foster belonging and sustained engagement.

The IPTs were refined through iterative team discussions involving researchers with expertise in behavioral science, digital health, and qualitative methods. Refinement emphasized theoretical coherence and cross-context relevance, particularly for LMIC settings where group chats are often the most accessible digital channel. Contextual barriers and facilitators were incorporated into a matrix accompanying the IPTs to support later analysis. The final framework, summarized in MultimediaAppendices 1  2, identified 6 SCT-informed pathways linking group chat mechanisms to outcomes: behavioral capability, self-efficacy, environmental affordances, observational learning, reinforcement, and self-regulation.

### Information Sources and Search Strategy

A comprehensive search of the peer-reviewed literature was conducted to identify empirical studies evaluating the use of group chat–based digital platforms as part of a health intervention. Initially, on September 19, 2025, searches were carried out in PubMed (via PubMed), Embase, MEDLINE (via Ovid), Web of Science (via Web of Science), and Scopus (via Scopus) for records published between January 1, 2005, and September 1, 2025, limited to English-language and human studies. Embase and MEDLINE were searched simultaneously on the Ovid platform (Wolters Kluwer) using a single search strategy. The search was rerun on February 18, 2026, to capture studies published from September 2, 2025, onward, using the same search concepts and eligibility criteria. Search strings combined controlled vocabulary and free-text terms related to group chats (eg, “group chat,” “WhatsApp group,” “Telegram group,” and “WeChat”) with health- and intervention-related terms (eg, “health promotion,” “behavior change,” “intervention,” “trial,” and “evaluation”). Filters excluded reviews, protocols, commentaries, and other nonempirical papers, and no published search filters were used. No additional restrictions were applied beyond the date range, English language, and human studies. The search strategy was not peer-reviewed and was not adapted from prior reviews. The complete search strategies for all databases, copied exactly as run (including the February 18, 2026 update), are provided in Checklist 2.

No study registries were searched, no targeted website browsing was undertaken, and no authors or experts were contacted to identify additional studies. Reference lists of included studies and relevant reviews were screened to identify additional publications, including linked articles reporting process evaluations or qualitative findings from the same intervention.

### Eligibility Criteria

The studies were eligible if they evaluated a health-related intervention in which a group chat served as the core digital space for intervention delivery and interaction. Group chat was defined as a multiuser messaging thread within a messaging platform that enables interaction among participants, with or without facilitation, via text and optionally images, audio, or video. Eligible interventions used the group chat to deliver one or more core functions such as health education, counseling or coaching, peer support, facilitated discussion, collaborative problem-solving, or care coordination, rather than logistics alone. Eligible study designs included randomized controlled trials (RCTs), quasi-experimental studies, pilot and feasibility studies, and program evaluations reporting quantitative, qualitative, or mixed methods findings. Included studies were mapped according to outcomes reported across behavioral, psychosocial, and clinical domains, as well as engagement and implementation outcomes where available. Studies reporting qualitative or process data linked to an intervention were retained where they contributed explanatory information to the realist synthesis.

Studies were excluded if they lacked a defined human population, did not implement a health intervention, or used group chats only peripherally. Peripheral use was defined as group chats used primarily for recruitment, administrative coordination, appointment scheduling, or 1-way announcements without meaningful interactive discussion or delivery of intervention content. Publications without primary data, including protocols, conceptual papers, reviews, and conference abstracts, were excluded. Only full-text, peer-reviewed journal articles were included.

### Study Selection

Records from all sources were imported into Covidence (Veritas Health Innovation), where duplicates were removed using the platform’s deduplication function with manual review to confirm and resolve remaining duplicates. To ensure consistent application of eligibility criteria, reviewers jointly piloted a small sample of abstracts prior to screening. Title and abstract screening and full-text screening were conducted independently by 2 reviewers. Disagreements were resolved through discussion, with a third reviewer consulted when consensus could not be reached. The study selection process is illustrated in the PRISMA (Preferred Reporting Items for Systematic Reviews and Meta-Analyses) flow diagram.

### Data Charting

Data charting was conducted using structured, piloted forms designed to extract and organize information for both the mapping of study characteristics and the realist synthesis components of the review. Data were extracted and entered into these forms by 1 reviewer and independently checked by a second reviewer, with discrepancies resolved through discussion and arbitration by a third reviewer when needed.

For the scoping review, charted items included bibliographic details (author and year), country or setting, health domain, study aims, study design, participant characteristics and sample size, platform type, intervention overview, and how the group chat functioned within the intervention. Where reported, we also charted key group chat features (eg, group structure, communication modalities, facilitation or moderation practices, and any stated privacy or participation rules), comparator conditions where applicable, outcome domains assessed, outcome measurement approaches and timing, and study-reported findings.

For the realist synthesis, reviewers extracted verbatim or summarized data describing contextual conditions (eg, cultural setting, group composition, and level of facilitation), mechanisms proposed or observed to drive change, and outcomes achieved. When mechanisms were not explicitly stated, they were inferred from the authors’ descriptions of processes or participant experiences. CMO coding was undertaken by 2 reviewers and refined through team discussion.

### Analysis and Synthesis

For the scoping review, extracted data were summarized descriptively to map study characteristics, intervention features, and outcome domains across the included studies. Studies were grouped by health domain, population, setting, and platform type, and outcomes were summarized across behavioral, psychosocial, and clinical domains, including how and when they were measured where reported. This descriptive synthesis was used to identify areas where evidence is more developed and where gaps remain.

For the realist synthesis, analysis followed a realist logic of inquiry to develop and refine program theories explaining how, why, and under what conditions group chat interventions achieved outcomes. The process began with all extracted CMO data, each describing how contextual features interacted with intervention processes to generate change. Using a reflexive approach to thematic analysis [35], the team examined CMOs to identify recurring patterns, interconnections, and configurations across studies. CMOs were coded and grouped into higher-order clusters representing common or complementary configurations observed across settings. As patterns stabilized, these clusters were organized into broader domains and compared against the IPTs. The developing domains were informed by, but not limited to, the original IPT categories. While SCT provided the conceptual scaffold, categorizations evolved where new mechanisms or linkages emerged, ensuring the final framework reflected both theoretical grounding and empirical diversity. The framework was iteratively refined through team discussion and consensus-building, with regular meetings to test interpretations, examine alternative explanations, and address negative cases. A detailed audit trail documented each iteration of coding, synthesis, and decision-making.

The final framework was distilled into a visual program theory that offered an evidence-based account of how group chat interventions operate and a heuristic for informing their design and adaptation. Relationship candidates were identified through cross-case comparison of CMO clusters and mapped only when supported by consistent empirical patterns or theoretically coherent linkages. The team iteratively refined these pathways through structured deliberation, examining rival explanations and negative cases to determine whether connections were salient enough to visualize. Directionality was assigned where temporal or causal ordering was evident in the data, while bidirectional relationships were included when reciprocal influence was repeatedly described.

## Results

### Included Studies

**Figure 1.**
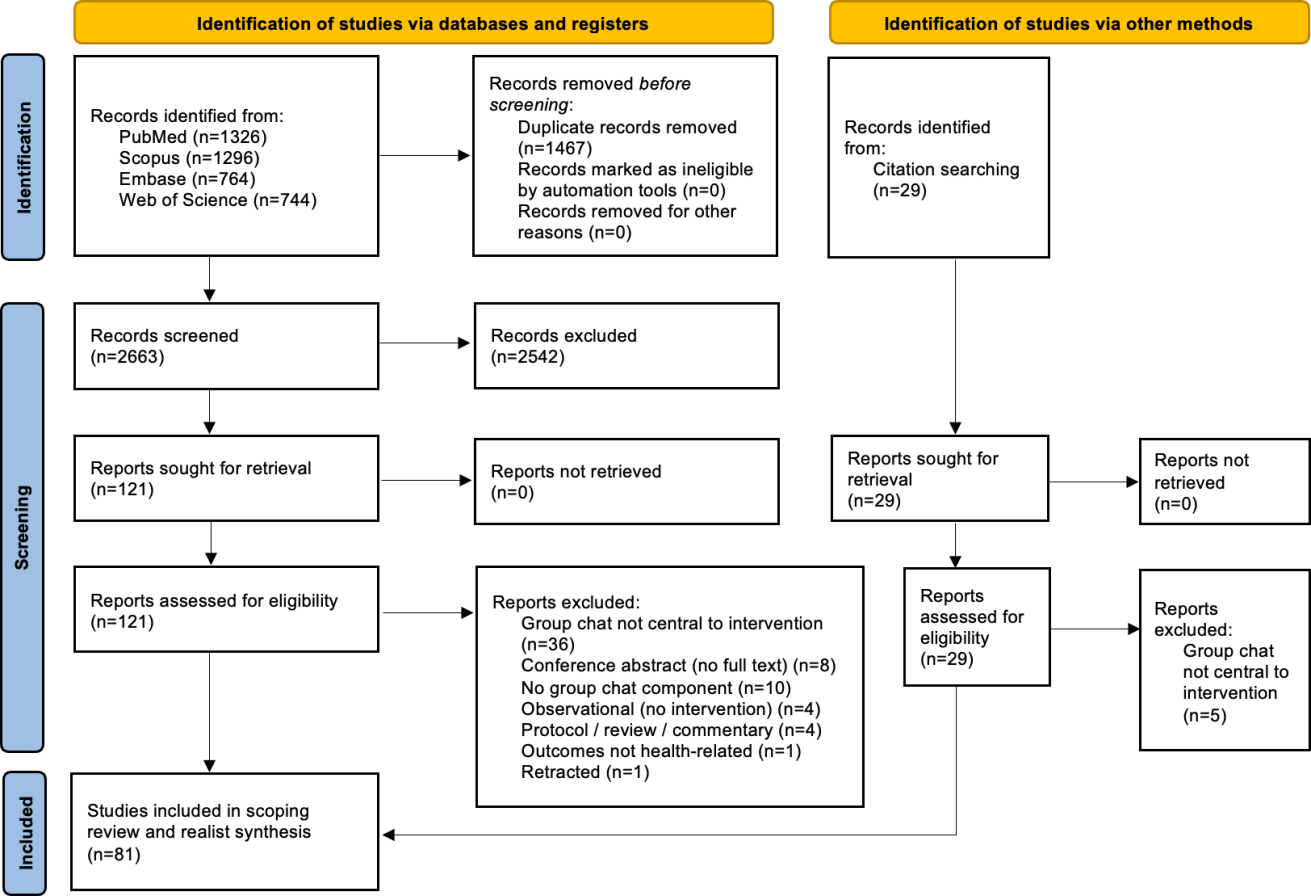
PRISMA (Preferred Reporting Items for Systematic Reviews and Meta-Analyses) diagram for studies included in the scoping review and realist synthesis of group chat interventions.

A total of 4130 records were identified through database searches (PubMed=1326; Scopus=1296; Embase=764; Web of Science=744) and manual citation screening (n=29). After removing 1467 duplicates, 2692 titles and abstracts were screened, of which 2542 were excluded. Full-text assessment was conducted for 150 articles, of which 69 were excluded because they did not meet the inclusion criteria, most commonly because group chat was not central to the intervention (n=41), no group chat component was present (n=10), full text was unavailable (n=8), or outcomes were not health related (n=1), with additional exclusions. A total of 81 studies [36-116] were included for the scoping review and realist synthesis (Figure 1).

### Study Characteristics

Table 1 summarizes the breadth of study designs, settings, and intervention features across the 81 included studies [36-116]. Designs spanned RCTs (30/81, 37%), quasi-experimental or single-group evaluations (15/81, 18.5%), and non-RCTs (11/81, 13.6%), alongside pilot and feasibility studies (10/81, 12.3%) and smaller numbers of qualitative (6/81, 7.4%), mixed methods (5/81, 6.2%), and program evaluations (4/81, 4.9%). Geographically, studies were distributed across 23 countries, with the largest single-country contributions from China (18/81, 22.2%) and the United States (10/81, 12.3%), followed by Indonesia (7/81, 8.6%), Hong Kong SAR and Iran (5/81 each, 6.2%), and Japan (4/81, 4.9%). Four studies were multicountry in design [41,61,78,86], and the studies spanned Sub-Saharan Africa, Latin America, the Middle East, Europe, and other parts of Asia (25/81, 30.9%).

**Table 1.** Summary of study characteristics.

Characteristics	Studies included (N=81)
Study design, n (%)	
Randomized controlled trial	30 (37.0)
Single-group or quasi-experimental study	15 (18.5)
Nonrandomized controlled trial	11 (13.6)
Pilot and feasibility study	10 (12.3)
Qualitative descriptive study	6 (7.4)
Mixed methods evaluation	5 (6.2)
Retrospective observational study	2 (2.5)
Real-world program evaluation	2 (2.5)
Country, n (%)	
China	18 (22.2)
United States	10 (12.3)
Indonesia	7 (8.6)
Hong Kong SAR	5 (6.2)
Iran	5 (6.2)
Japan	4 (4.9)
Multicountry	4 (4.9)
Germany	3 (3.7)
Other^a^	25 (30.9)
Health topic, n (%)	
Mental health	25 (30.9)
Maternal and child health	20 (24.7)
Weight management, nutrition, or eating disorders	13 (16.0)
Smoking cessation	11 (13.6)
Dementia and caregiving	9 (11.1)
Diabetes	8 (9.9)
Physical activity	7 (8.6)
Others^b^	37 (45.7)
Setting^c^, n (%)	
Clinical	39 (48.1)
Community or home	34 (42.0)
Workplace or school	8 (9.9)
Type of membership, n (%)	
Mixed membership	68 (84.0)
Homogeneous peer-led	13 (16.0)
Communication modalities^d^, n (%)	
Text+dynamic media (audios, videos, etc)	41 (50.6)
Text only	20 (24.7)
Text+static media (images, emojis, links, etc)	15 (18.5)
Not explicitly stated	5 (6.2)
SCT^e^ constructs aligned, n (%)	
Behavioral capability	61 (75.3)
Self-efficacy	63 (77.8)
Environment	69 (85.2)
Observational learning	58 (71.6)
Reinforcements	70 (86.4)
Self-control	22 (27.2)
Theories explicitly connected, n (%)	
Cognitive behavioral therapy	4 (4.9)
Social cognitive theory	7 (8.6)
Health Belief Model	3 (3.7)
Other^f^	21 (25.9)
Importance of group chat as intervention^g^, n (%)	
Core intervention	42 (51.9)
Standalone intervention	13 (16.0)
Reinforcement intervention	26 (32.1)

a The other countries include Kenya, Turkey, Cameroon, Uganda, Brazil, South Africa, Ghana, Nigeria, Tanzania, United Kingdom, Italy, Canada, South Korea, Saudi Arabia, Netherlands, and Philippines.

b The other clinical topics include cancer and oncology, chronic disease self-management, parenting and child development, immunization, sexual and reproductive health, respiratory diseases, substance use, and rehabilitation.

c For setting, clinical interventions or interventions between health professionals were chosen as “clinical,” public health interventions which happened at the community level were chosen as “community/home” and workplace or school-based interventions were chosen as “workplace/school.”

d The chat platforms included WhatsApp, WeChat, Line, Viber, KakaoTalk, and Telegram.

e SCT: social cognitive theory.

f The other theories explicitly connected include Social Learning Theory, Parenting for Lifelong Health framework, Haddon Matrix, locus of control theory, Big Five Theory, MOST (Multiphase Optimization Strategy Framework) framework, Theory of Reasoned Action, Behavior Change Techniques (BCTs) taxonomy, Theory of Gender and Power, Information-Motivation-Behavior (IMB) Model, Behavior Change Wheel (BCW), Socioecological model, Theoretical framework of Acceptability, Psychoanalytic/Small Group theory, Wagner’s Chronic Illness Care (CIC) Model, Information-Motivation-Behavioral Skills (IMB) theory, Inquiry-Based Learning (IBL), Reinforcement theory, Capability, Opportunity, Motivation–Behavior (COM-B) Theory, Planned Behavior Theory, Self-Regulation Theory, Self-Discrepancy Theory, and Peers for Progress framework.

g If the group chat is: major part of the whole intervention for behavioral change, it is rated as “core”; the one and only behavioral intervention, it is rated as “standalone”; and as reinforcement or supplementary to the other behavioral interventions, it is rated as “reinforcement.”

The health topics addressed were diverse and often overlapping across studies. Mental health was the most frequently targeted domain (25/81, 30.9%), encompassing interventions addressing depression, anxiety, caregiving burden, and related psychosocial conditions. Maternal and child health was the second most common focus (20/81, 24.7%), including antenatal and postnatal care, breastfeeding support, and child development programs. Other prominent topics included weight management, nutrition, or eating disorders (13/81, 16%), smoking cessation (11/81, 13.6%), dementia and caregiving (9/81, 11.1%), diabetes self-management (8/81, 9.9%), and physical activity (7/81, 8.6%). A heterogeneous range of topics was addressed (37/81, 45.7%), including HIV, cancer, cardiovascular rehabilitation, and chronic obstructive pulmonary disease self-management. As studies frequently addressed more than 1 health domain, these proportions sum to more than 100%.

Nearly half of the studies were implemented in clinical settings (39/81, 48.1%), with the remainder primarily in community or home settings (34/81, 42%) and a smaller subset in workplace or school settings (8/81, 9.9%). Most interventions used mixed membership group formats comprising health professionals alongside participants (68/81, 84%) rather than homogeneous peer-led groups (13/81, 16%). Communication modalities were most often text combined with dynamic media such as audio and video (41/81, 50.6%), followed by text-only (20/81, 24.7%) and text combined with static media such as images, emojis, and links (15/81, 18.5%). Group chats most often served as the core intervention component (42/81, 51.9%), followed by use as a reinforcement or adjunct within broader multicomponent programs (26/81, 32.1%), and as standalone interventions (13/81, 16.0%). Of the 81 studies [36-116], theoretical frameworks connected to interventions included SCT (n=7, 8.6%), cognitive behavioral therapy (n=4, 4.9%), and the Health Belief Model (n=3, 3.7%). SCT constructs were commonly identifiable across interventions, with reinforcements (70/81, 86.4%) and environment (69/81, 85.2%) most frequently operationalized, followed by self-efficacy (63/81, 77.8%), behavioral capability (61/81, 75.3%), and observational learning (58/81, 71.6%). These descriptive patterns supported later theory-informed synthesis rather than serving as causal claims within the mapping stage.

**Figure 2.**
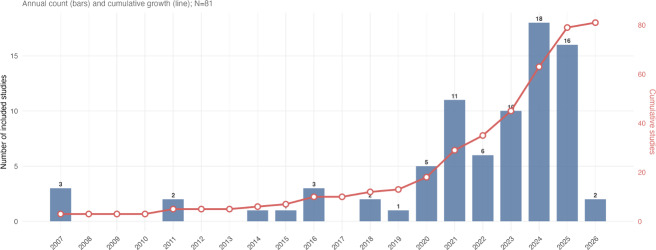
Trends of the included publications over time (N=81).

The publication timeline indicates a small number of early studies from 2007 onward, followed by accelerated growth in the early 2020s (Figure 2). More than three-quarters of the evidence base (63/81, 77.8%) was published between 2020 and 2025, with the cumulative total increasing sharply in recent years. Prior to 2020, annual publication counts remained low, with no more than 3 studies published in any single year. This figure is intended to situate the field’s maturity and pace of expansion rather than to infer drivers of growth.

### Distribution of Platforms, Settings, and Evidence Across Health Topics

**Figure 3.**
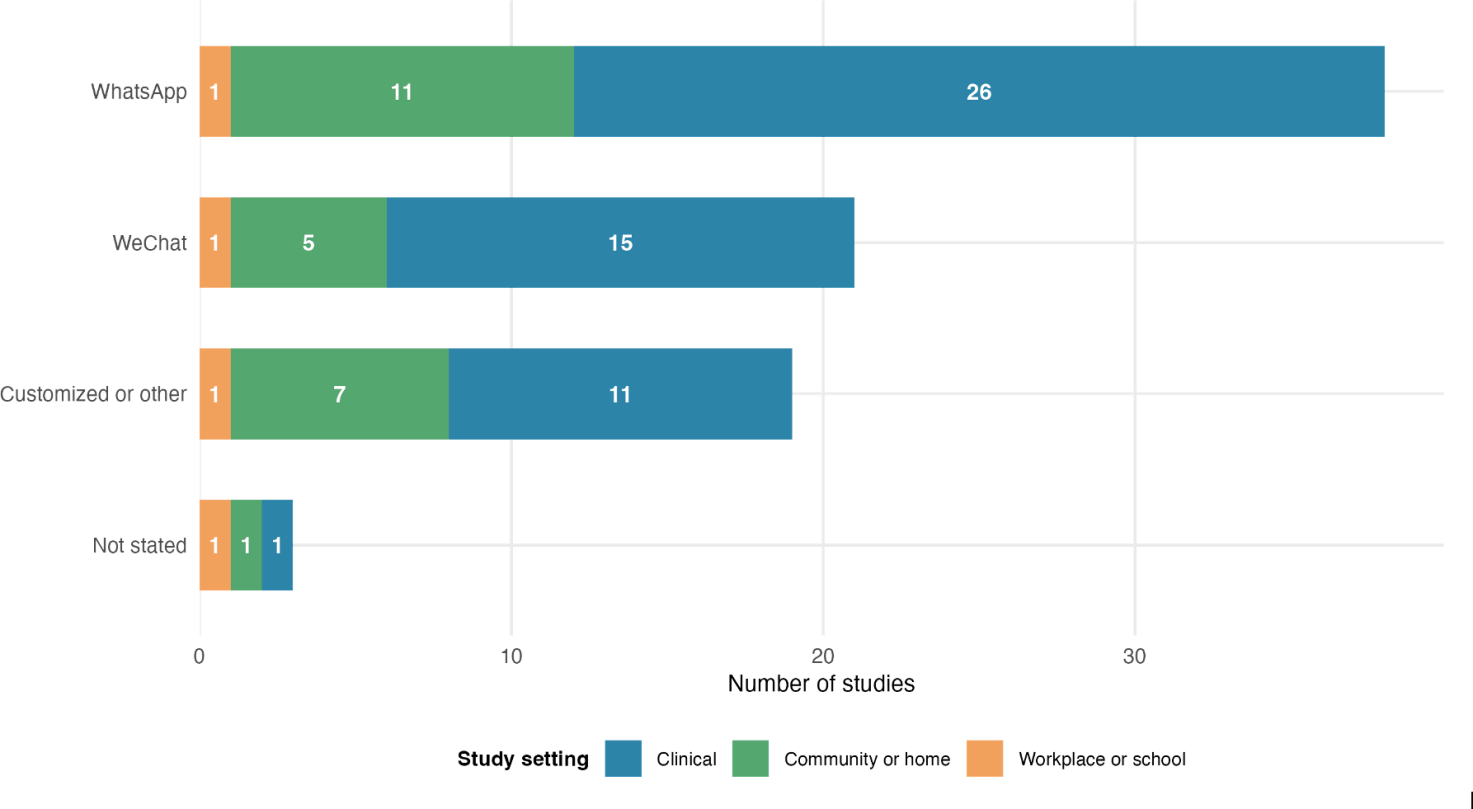
Usage of group chat platforms by study settings (N=81). The other chat platforms include Line, Viber, KakaoTalk, and Telegram.

Platform use varied by context (Figure 3). WhatsApp was the most frequently studied platform overall (38/81, 46.9%), with substantial representation in community settings (n=26) and additional use in clinical settings (n=11). WeChat was also commonly evaluated (21/81, 25.9%), largely in clinical contexts (n=15), reflecting its integration within China’s health care system. Customized or other platforms, including LINE, Viber, KakaoTalk (Kakao Corporation), and Telegram, were used across both clinical and community settings (19/81, 23.5%). This mapping is presented to describe delivery channels across contexts and to support later interpretation of how platform affordances may shape implementation conditions.

**Figure 4.**
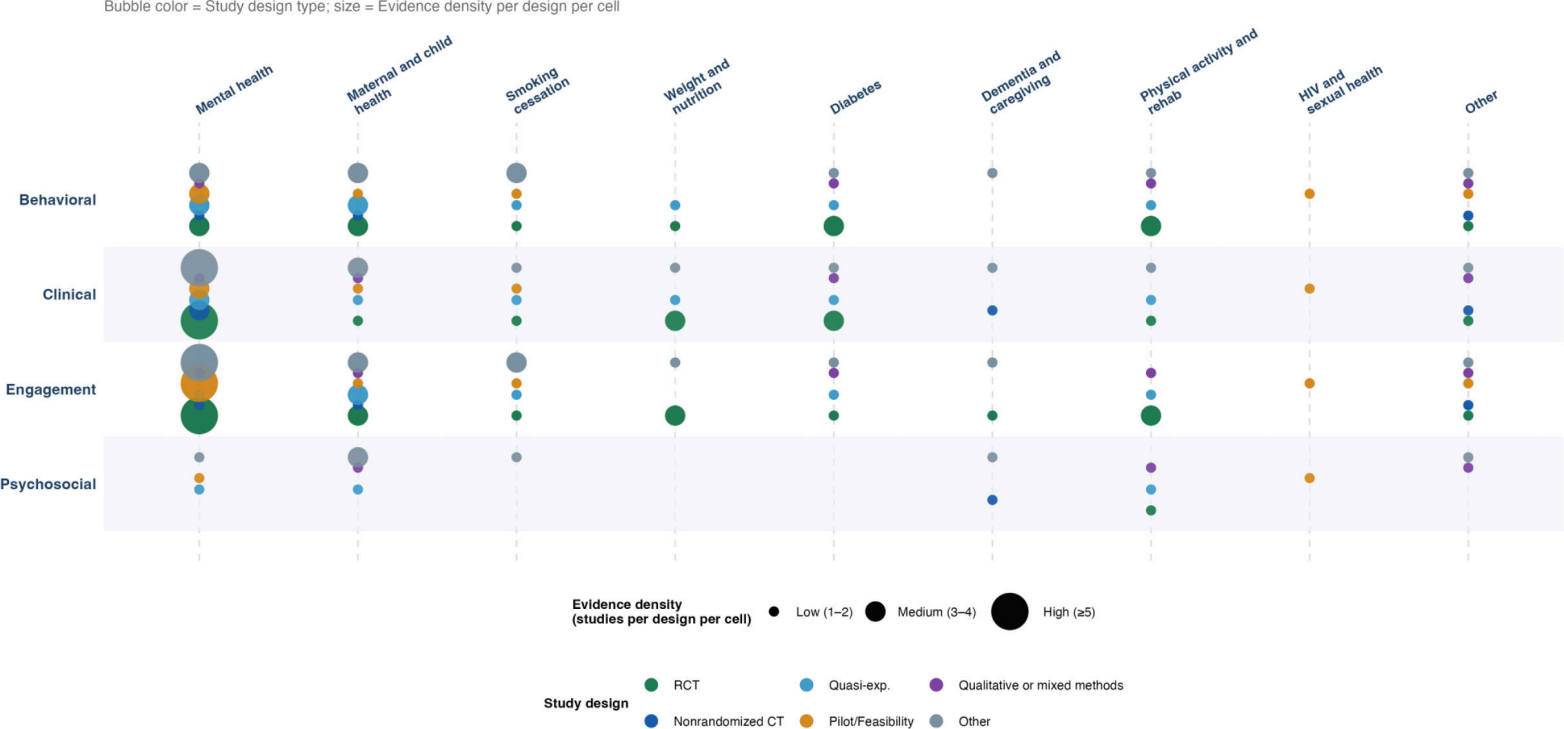
Evidence gap map of group chat interventions based on outcome domains and clinical topics (N=81). Others in the study design include mixed methods evaluation, retrospective observational study, and real-world program evaluation. Other in the clinical topics include cancer and oncology, chronic disease self-management, parenting and child development, immunization, respiratory diseases, substance use, and rehabilitation. RCT: randomized controlled trial.

To further characterize the distribution of evidence across health topics and outcome domains, Figure 4 presents an evidence gap map of all 81 included studies [36-116]. Engagement and behavioral outcomes were the most consistently studied across nearly all health topics, while psychosocial outcomes remained notably sparse, particularly for smoking cessation, weight management, and dementia and caregiving, indicating areas where the evidence base requires further development.

### Final CMO Configurations

The analysis produced 12 CMO configurations, reflecting the most recurrent and explanatory patterns across the included studies. These configurations describe how specific contextual conditions triggered mechanisms within group chat–based interventions, shaping behavioral, psychosocial, and engagement outcomes (Multimedia Appendix 3) [36,37,39,40,42-47,49-54,58,59,61-63,65-69,71,72,74,75,77,79,81,82,84-86,88-90,94-96,99-106,108-116].

#### Domain 1: Capability and Actionability

Group chats enhanced participants’ capacity to adopt health behaviors through several mechanisms. Timed prompts broke down complex actions into manageable steps, aiding timely task completion; for example, a WhatsApp program in Semarang used frequent prompts and multimedia guidance to support early breastfeeding, iron use, hygiene, and danger-sign monitoring [100]. Multimedia content (eg, videos and audio clips) paired with threaded Q&As helped participants develop practical skills by allowing them to view demonstrations, ask questions, and revisit materials. Chats also corrected misinformation in real time, reducing cognitive dissonance, such as in a Hong Kong WhatsApp group where mothers clarified details about influenza vaccination and gained confidence in clinic navigation [71]. These elements collectively strengthened behavioral capability across varied health domains.

#### Domain 2: Confidence and Motivation

Sustaining participants’ belief in their capacity to change relied on personalized encouragement, visible recognition, and synchronous interaction. Tailored moderator feedback helped participants reframe setbacks constructively, boosting confidence and activating peer accountability. For example, a WhatsApp-based cognitive behavioral therapy program for overweight women in Iran used daily therapist interactions to support cognitive reframing and behavior monitoring, improving BMI [36]. Group recognition through photo sharing, comments, and public acknowledgment amplified motivation by making progress visible, fostering social validation and sustained engagement. Some interventions added brief audio or video calls, enhancing text-based support with immediacy and nonverbal cues. For instance, weekly video-based group chats that used real-time audio and visible nonverbal cues created stronger social presence and cohesion, leading to higher engagement compared with text-only formats [98]. This synchronous interaction deepened interpersonal bonds, increasing accountability and mutual support.

#### Domain 3: Modeling and Norms

Observational learning worked through direct peer exchange and the emergence of informal knowledge brokers. When participants shared personal experiences and outcomes, others gained concrete, relatable examples of applying health behaviors in real-life contexts. This peer demonstration normalized both success and struggle. For example, shared contraceptive experiences in digitally facilitated postabortion groups reduced anxiety and boosted confidence in effective method adoption [85]. Social comparison helped participants identify feasible tactics and adjust expectations based on peers’ experiences. In some groups, participants assumed bridging roles, especially when expert input was intermittent. These emergent peer leaders relayed information, validated insights, and sustained discussion, reducing the gap between laypeople and professionals. For example, in moderated parent groups, more knowledgeable members absorbed expert input and began clarifying information and guiding peers [53]. These informal knowledge brokers multiplied learning pathways and strengthened network cohesion, enhancing the sustainability of professional guidance.

#### Domain 4: Safe, Supportive Environment and Access

Group chat effectiveness hinged on structural and social characteristics that fostered authentic engagement. Small, closed groups with privacy safeguards and clear participation rules created psychological safety, encouraging participants to disclose sensitive information. For instance, anonymous online chats for adolescents with depressive symptoms reduced judgment fears and deepened emotional sharing [54]. However, privacy alone was insufficient; supportive social norms that discouraged judgment and promoted mutual respect were also key. Cultural and linguistic alignment further shaped trust and relevance. Interventions using participants’ primary language, culturally familiar content, and trusted messengers enhanced understanding and engagement. For example, a WeChat cessation program for Chinese immigrant smokers used tailored messages and coach support to improve quitting knowledge and self-efficacy [65]. Such cultural fit was especially crucial for low-literacy or marginalized communities often overlooked by standard health communication.

#### Domain 5: Self-Regulation and Maintenance

Group chats sustained behavior change by externalizing accountability and automating behavioral cues. Public goal setting with daily status updates made commitments visible, fostering social accountability and self-monitoring. Peer visibility created gentle pressure to follow through and modeled consistent behavior, as seen in small anonymous digital groups where daily abstinence updates and peer “applause” improved motivation and self-efficacy, leading to higher smoking cessation rates at 12 weeks [102]. Frequent peer messages reinforced shared goals through consistent social prompting. Time-based reminders complemented this by serving as environmental cues, reducing reliance on fluctuating motivation and helping establish habitual routines. A WeChat program for coronary artery disease patients used thrice-weekly messages for 12 months to reinforce self-management and improve diet, exercise, medication adherence, and blood pressure monitoring [67]. Despite these insights, evidence on the persistence of these self-regulation mechanisms after interventions ended remains limited, leaving long-term maintenance uncertain.

### Consolidated Program Theory of Group Chat Interventions

**Figure 5.**
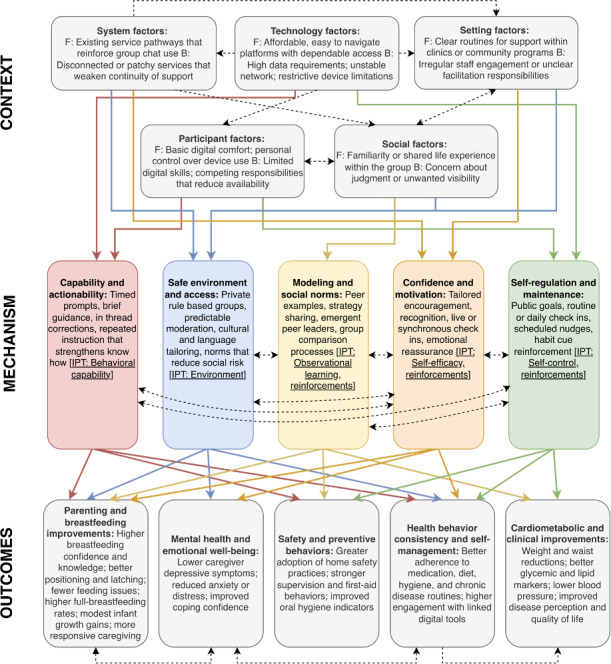
Visualized context-mechanism-outcome pathways from realist synthesis and scoping review describing how group chat interventions were observed to shape health-related outcomes. The figure synthesizes cross-study context-mechanism-outcome patterns into a final program theory. Contextual conditions (system, technology, setting, participant, and social factors) list facilitators (F) and barriers (B). Colored solid arrows represent the primary pathways underpinning the 5 major domains in the Final Program Theory (Multimedia Appendix 3) [36,37,39,40,42-47,49-54,58,59,61-63,65-69,71,72,74,75,77,79,81,82,84-86,88-90,94-96,99-106,108-116] and their most salient context-mechanism-outcome relationships. Dotted arrows indicate additional relationships within or across context, mechanism, or outcome domains. One-way arrows depict primarily unidirectional influences, while 2-way arrows reflect bidirectional or mutually reinforcing relationships. Outcome clusters summarize the main proximal and distal effects derived from the scoping review. Arrows visualize the most prominent patterns identified, while other secondary or more distal relationships may exist. The figure highlights those most consistently supported across included studies.

Building on the CMO domains in Multimedia Appendix 3 [36,37,39,40,42-47,49-54,58,59,61-63,65-69,71,72,74,75,77,79,81,82,84-86,88-90,94-96,99-106,108-116], Figure 5 synthesizes how group chat interventions were observed to operate within layered and interacting contextual conditions. Rather than functioning within single, isolated enablers, systems, technology, settings, participants, and social factors often combined in ways that shaped who participated, how safely they engaged, and how reliably groups were maintained. Across studies, conditions that supported digital access, regular facilitation, and a sense of social familiarity or safety frequently co-occurred, while technological instability, limited staff presence, or socially constrained dynamics were commonly described as barriers.

Within these contextual environments, the mechanism patterns identified in the review tended to operate in combination. Capability-building processes (eg, prompts, guidance, and corrections) were often noted alongside elements that created a psychologically safe space for questions or disclosure. Peer modeling and emerging norms helped translate guidance into everyday routines, while motivational inputs and emotional reassurance supported continued effort. Self-regulation processes, such as routine check-ins, public goals, and habit cues, were typically described in studies where other mechanisms were already active. Across interventions, these interdependencies appeared to shape how participants learned, interacted, and sustained behavioral changes over time.

The outcome layer of the model draws on the scoping review and links these mechanisms to behavioral, psychosocial, and clinical outcomes. Improvements were reported in breastfeeding technique and caregiving practices, safety and hygiene behaviors, emotional well-being, and day-to-day disease self-management. Across studies, health behavior consistency and self-management routines emerged as an important foundation for other outcomes, frequently appearing alongside or prior to changes in clinical indicators such as weight, glycemic markers, blood pressure, or disease perceptions. Examples from the review illustrate these patterns: in clinic-based WhatsApp cessation groups in Hong Kong, recent quitters who engaged more consistently benefited from peer reinforcement and achieved higher abstinence rates [57], whereas in maternal health groups where husbands felt embarrassed and participation remained low, limited psychological safety and weak norms were noted as barriers to attitudinal change [100]. Overall, the program theory depicts group chat interventions as dynamic, socially embedded systems whose effectiveness depends on how well contextual conditions, mechanisms, and outcomes remain in alignment over time.

## Discussion

### Principal Findings

This scoping review with realist synthesis mapped and explained the evidence on group chat–based health interventions. In line with our objectives, the scoping review component described where evidence is concentrated and how interventions have been designed and evaluated, while the realist synthesis examined how contextual conditions shape engagement and behavior change. Findings highlight how the evidence base on group chat interventions has grown rapidly since 2020 and is concentrated most strongly in mental health and maternal and child health. WhatsApp and WeChat were the most commonly used platforms across community and clinical settings, and most interventions relied on mixed membership groups that included health professionals alongside participants. The realist synthesis identified 12 recurring CMO configurations that clustered into 5 mechanism domains: capability and actionability, confidence and motivation, modeling and norms, safe supportive environments and access, and self-regulation and maintenance. Together, these findings offer a theory-informed account of how group chat interventions operate in practice and highlight key evidence gaps, including the underrepresentation of older adults and lower literacy users and the limited reporting of implementation processes.

In many digital health programs, group chats are positioned as an enabling social layer alongside other components (eg, coaching sessions, educational modules, wearables, or reminder systems), which may enhance support but also make it difficult to isolate whether observed effects are driven by peer processes, professional input, self-monitoring, or their interaction. We also excluded many studies where group chats were mentioned but not substantively implemented. In several cases, the feature was added with minimal detail on facilitation, engagement, or theoretical rationale, suggesting it was treated as a generic communication channel rather than a mechanism-based component. Taken together, these patterns indicate a broader missed opportunity: group chats are often positioned as supplementary add-ons rather than designed and evaluated as interventions with their own distinct social and behavioral mechanisms.

Previous reviews on digital health communications, including those in digital mental health, tend to emphasize adaptability, cost, and organizational policies rather than the mechanisms that drive behavior change [13]. At the same time, earlier realist reviews often pool together diverse digital health technologies [12,117], which makes it difficult to detect modality-specific dynamics. By focusing specifically on group chat interventions, our review is able to surface mechanisms such as peer modeling, emergent leadership, and collective accountability that are overlooked in broader syntheses. We further show that informal peer leaders and knowledge brokers naturally emerge within these groups [46,53,68,101], suggesting that long-term effectiveness may depend more on peer-to-peer support than on continuous professional involvement. This contrasts with Schlief et al [118] emphasis on therapeutic relationships and Shahid et al [119] focus on team-based professional care, highlighting how the peer-led potential of group chats remains underused. Moreover, the predominance of group chat interventions in LMICs, especially in Asia, was another notable finding. Rapid mobile uptake [120], collectivist social norms [121], and rising chronic disease burdens [122,123] create strong demand for low-cost, scalable tools that mirror everyday communication practices. These contexts also underscore the potential of group chats to reach users who may be excluded by app-centric or data-heavy models [124], including those with shared devices, limited data, or intermittent access. At the same time, a clear disconnect emerged: although group chats are widely used by populations often absent from more complex digital health tools (including older adults and lower literacy users), these groups were still largely missing from the intervention studies. This likely reflects recruitment patterns centered on maternal, youth, and clinic-based populations rather than the wider pool of everyday messaging app users [125,126]. A related concern is that recruitment may still be shaped by assumptions about which groups are easiest to reach digitally, even though recent participatory realist work cautions against assuming who can or cannot benefit without empirical evidence [127]. Broadening future studies to include these underrepresented groups and testing needed adaptations in facilitation, content, or onboarding will be essential for understanding how group chat interventions function across diverse ages, literacy levels, and access conditions [12].

The group chats in our realist synthesis were predominantly small, closed groups with preexisting relationships, intensive facilitation by providers or researchers, and a single, bounded communication space [36,44,46,54,58,61,69,72,74,81,89,100,101,103,114-116]. These design choices supported safety and disclosure but limited the multidirectional information flows across wider social networks that naturally occur in everyday messaging use [127]. By contrast, observational work on platforms such as WhatsApp, WeChat, and Facebook Messenger (Meta Platforms, Inc) has shown that group chats can rapidly diffuse information and misinformation during crises [128], coordinate mutual aid [129], and function as hubs for resource sharing and collective problem-solving [130]. Across intervention trials, effects also often appeared to be contingent on engagement intensity and facilitation quality, reinforcing that group chats often operate as social and motivational infrastructures rather than simple content-delivery mechanisms [131]. Our realist synthesis highlighted the centrality of peers in driving engagement and change within intervention groups [37,43,44,46,52,53,59,61,68,69,75,81,82,84,85,88-90,94,95,101-104,108-110,113-116], suggesting substantial untapped potential to design group chat interventions that position communities as active agents of change rather than passive recipients. Future work could more deliberately harness social diffusion, for example, by seeding core groups and explicitly supporting participants to adapt and carry content into their own family, neighborhood, faith-based, or workplace chats, while safeguarding norms of privacy and trust. Some emerging models already point in this direction. For instance, the “Let’s Chat” intervention trained young Vietnamese American adults as family health advocates who then initiated and led cancer screening discussions within their existing intergenerational family group chats, demonstrating how a relatively light-touch scaffold can catalyze preventive conversations across multiple related networks [46]. Designing and testing similar networked group chat architectures, including multigroup structures and clearly defined bridging roles, represents a key opportunity for innovation in digital health.

This review has several strengths, including being the first to focus specifically on group chats as a primary digital health delivery channel, integrating a scoping review of empirical studies with a realist synthesis grounded in an a priori SCT-informed program theory and conducted in line with PRISMA-ScR [30] and RAMESES II [32]. This methodological combination provides a unique contribution by mapping how group chat interventions have been designed and implemented across settings while also explaining the mechanisms and contextual conditions through which they operate in practice. Our findings show that group messaging functions as a socially embedded environment in which capability building, modeling, reinforcement, motivation, and self-regulation unfold through everyday interaction. The realist approach was especially valuable for unpacking these processes, allowing us to identify recurring CMO patterns across diverse platforms and settings. These insights advance behavioral theory in digital health by showing how constructs such as observational learning, collective efficacy, and social reinforcement are activated within group chat ecosystems, and they offer clearer design principles for tailoring interventions to local cultures, facilitation capacities, and user needs.

### Limitations

Several limitations should be noted. First, a formal critical appraisal across all included studies was not undertaken. Given the heterogeneity in study designs, populations, intervention formats, and outcome measures, applying a single quality assessment tool across the full evidence base was not feasible. Second, we limited inclusion to peer-reviewed, English-language full texts; this language restriction may have excluded relevant studies and may bias the apparent geographic distribution of evidence, particularly for platforms and settings where local language publication is common. We also excluded conference proceedings, protocols, and gray literature, which may have led to undercapture of emerging interventions and implementation learnings that are not yet published as full articles. Third, realist inference depends on the availability of contextual and process data, yet many primary studies, including RCT reports, provided limited detail on facilitation practices, engagement dynamics, and implementation adaptations. The frequent absence of rich process data constrained CMO specification in some cases and may have required cautious inference from brief descriptions, potentially leading to underspecification or misclassification of mechanisms and contexts.

### Conclusion

Group chats are central to how communities communicate yet remain underused in formal digital health design. This review shows that group chats are not only delivery channels but social infrastructure that can mobilize collective action, reinforce norms, and support sustained behavior change in ways distinct from other digital modalities. By centering group chats as a distinct intervention format and integrating a scoping review with realist program theory, we explain how context and facilitation activate mechanisms of change, offering a practical roadmap for designing and scaling low-cost, high-reach interventions within broader digital initiatives. AI-enabled chatbots and facilitation tools may extend this potential through scalable tailoring, just-in-time prompts, multilingual clarification, and triage while reducing moderator burden [111], but also raise design and governance questions around trust, peer dynamics, misinformation and safety, and when human facilitation is essential for relationship-building and accountability [132]. Compared with prior reviews that pool digital modalities or focus primarily on effectiveness [20,22,24], this synthesis clarifies what makes group chats distinctive and actionable for implementation.

In practice, group chat interventions may be most useful when deliberately structured to harness peer interaction and facilitation, or when integrated with complementary modalities such as brief onboarding or coaching to establish goals and norms [133], self-guided app or web modules for skill building reinforced through chat-based practice and feedback [134], and SMS or wearable-triggered prompts that feed into timely group problem-solving and accountability [135]. The program theory generated here supports implementers and policymakers in configuring design features, facilitation approaches, and contextual conditions (system, technology, setting, participant, and social) to activate specific mechanisms, moving beyond ad hoc use of group message threads toward purpose-built group environments. For researchers, priorities include targeted evaluations of chatbot-mediated group chat interventions, testing how agents deliver core functions (prompting, feedback, personalization, and triage) within groups, and comparative studies that isolate the contribution of group chat components relative to other digital modalities. With deliberate, theory-driven development, group chat interventions can be more consistently specified, implemented, and evaluated as a scalable and equitable component of digital health systems across diverse settings.

## Supplementary material

10.2196/88911 Multimedia Appendix 1 Initial Program Theories for group chat–based health interventions (guided by social cognitive theory).

10.2196/88911 Multimedia Appendix 2 Cross-cutting facilitators and barriers for activating Initial Program Theory mechanisms in group chat–based interventions.

10.2196/88911 Multimedia Appendix 3 Final program theory synthesizing context-mechanism-outcome configurations in group chat health interventions was analyzed.

10.2196/88911 Checklist 1 PRISMA-ScR checklist.

10.2196/88911 Checklist 2 PRISMA-S checklist.

## Abbreviations:

AI: artificial intelligence
CMO: context-mechanism-outcome
IPT: Initial Program Theory
LMIC: low- and middle-income country
PRISMA: Preferred Reporting Items for Systematic Reviews and Meta-Analyses
PRISMA-S: Preferred Reporting Items for Systematic Reviews and Meta-Analyses literature search extension
PRISMA-ScR: Preferred Reporting Items for Systematic Reviews and Meta-Analyses extension for Scoping Reviews
RAMESES II: Realist And Meta-narrative Evidence Syntheses: Evolving Standards II
RCT: randomized controlled trial
SCT: social cognitive theory
