# Supplementary material for: Using Group Chats to Drive Behavior Change in Digital Health Interventions: Scoping Review and Realist Synthesis
Source: J Med Internet Res. 2026 Apr 13;28:e88911. doi: 10.2196/88911 (PMC13075640; doi:10.2196/88911)
Supplement: Multimedia Appendix 1 [file jmir-v28-e88911-s001.docx]

**Initial Programme Theories (IPTs) for group chat–based health interventions (guided by Social Cognitive Theory)**

| **SCT Construct** | **Context (C)** | **Mechanism (M)** | **Outcome (O)** |
| --- | --- | --- | --- |
| **Behavioral Capability** | The group chat serves as a main space for sharing clear and practical health information, including explanations, examples, and step-by-step guidance from facilitators or trusted peers | Consistent exposure to simple, relevant, and repeated information helps participants understand what actions are needed and how to do them correctly | Improved knowledge and skills to carry out target health behaviours |
| **Self-Efficacy** | Facilitators and peers use the chat to provide encouragement, problem-solving support, and feedback on participants’ own progress or efforts | Personalised guidance and recognition increase participants’ belief in their ability to make and sustain changes, even when challenges arise | Greater confidence and persistence in practising health behaviours |
| **Environment** | The chat platform creates an ongoing, low-barrier setting where participants can connect easily, exchange messages freely, and feel supported by peers and facilitators | The sense of inclusion, continuity, and responsiveness makes people feel comfortable engaging and staying active in discussions | Higher levels of participation, regular communication, and retention over time |
| **Observational Learning** | Participants can see how others in the group describe their actions, adjustments, and outcomes related to behaviour change | By watching peers’ real experiences, participants learn what works, compare strategies, and adapt behaviours that seem achievable in their own context | Uptake and spread of effective health practices across members |
| **Reinforcements** | Positive feedback and visible acknowledgment of participation or achievements are shared openly within the chat | Social approval and recognition make participation rewarding and signal that certain actions are valued | Continued engagement, stronger motivation, and reinforcement of positive norms |
| **Self-Control** | The group includes regular opportunities for members to share goals, track progress, and reflect on what has or hasn’t worked | Self-monitoring and reflection within a supportive setting strengthen accountability and help participants adjust behaviour over time | Improved consistency, habit formation, and long-term maintenance of healthy behaviours |
