# Supplementary material for: Using Group Chats to Drive Behavior Change in Digital Health Interventions: Scoping Review and Realist Synthesis
Source: J Med Internet Res. 2026 Apr 13;28:e88911. doi: 10.2196/88911 (PMC13075640; doi:10.2196/88911)
Supplement: Multimedia Appendix 2 [file jmir-v28-e88911-s002.docx]

**Cross-cutting facilitators and barriers for activating IPT mechanisms in group chat–based interventions**

| **Type** | **Factor** | **How it affects IPT activation in group chats** | **IPT mechanisms most influenced** |
| --- | --- | --- | --- |
| Facilitator | Consistent, role-clear facilitation | Increases the likelihood that IPT mechanisms are triggered by keeping exchanges purposeful, resolving queries, and signalling credible support | Behavioral Capability; Self-efficacy; Reinforcements; Environment |
| Facilitator | Clear participation norms & privacy assurances | Lowers thresholds for disclosure and interaction, enabling mechanisms to operate within a safe, predictable social frame | Environment; Self-efficacy; Self-control |
| Facilitator | Contextualised, actionable content | Aligns guidance with lived contexts so capability-related mechanisms are activated and translated into practice | Behavioral Capability; Self-control |
| Facilitator | Appropriate group topology | Maintains visibility and relevance of contributions, strengthening modelling and engagement pathways central to the IPT | Observational Learning; Environment; Self-efficacy |
| Facilitator | Feedback & recognition culture | Makes reinforcement cues salient in-thread, sustaining motivation and normalising desired actions posited in the IPT | Reinforcements; Self-efficacy; Environment |
| Facilitator | Embedded self-monitoring structures | Creates regular occasions for goal articulation and reflection, activating self-regulation pathways specified by the IPT | Self-control; Reinforcements |
| Barrier | Misinformation or conflicting advice | Disrupts IPT activation by degrading credibility signals and confusing capability and efficacy pathways | Behavioral Capability; Observational Learning; Self-efficacy |
| Barrier | Message overload and topic drift | Obscures actionable content and interrupts conversational continuity required for IPT mechanisms to operate | Environment; Self-control; Reinforcements |
| Barrier | Participation concentration | Limits diversity and relatability of models, weakening observational learning and efficacy formation in the IPT | Observational Learning; Environment; Self-efficacy |
| Barrier | Temporal fragmentation | Delays or breaks feedback loops, reducing the timeliness needed for reinforcement and regulation within the IPT | Environment; Self-control; Reinforcements |
| Barrier | Stigma-sensitive content | Suppresses visible sharing that underpins modelling and confidence-building pathways described in the IPT | Observational Learning; Self-efficacy; Environment |
| Barrier | Access and digital inequities | Constrains basic participation, limiting exposure to the interactional conditions required for IPT mechanisms | Environment; Behavioral Capability; Self-control |
