# Supplementary material for: Using Group Chats to Drive Behavior Change in Digital Health Interventions: Scoping Review and Realist Synthesis
Source: J Med Internet Res. 2026 Apr 13;28:e88911. doi: 10.2196/88911 (PMC13075640; doi:10.2196/88911)
Supplement: Multimedia Appendix 3 [file jmir-v28-e88911-s003.doc]

**Final program theory synthesizing CMO configurations in group chat health interventions was analyzed.**

| **Domain** | **CMO title** | **CMO description** | **Study citations (Type of evidence)** | **CMO examples from included studies** |
| --- | --- | --- | --- | --- |
| Domain 1: Capability & Actionability (IPT: Behavioral Capability) | CMO 1.1 — Timed step-wise prompts improve on-time action | In group chats that deliver cadenced, contextual, step-by-step guidance (C), participants build practical know-how (M) and perform target actions on time (O). | [37,42,49-51,62,63,65-67,71,86,96,100,101,106,109,111-113]  (Randomized controlled trial, Single group/Quasi-experimental study, Pilot/Feasibility study, Mixed methods evaluation) | In contexts where counselors shared structured, relapse-prevention reminders 3 times weekly and prompted members to discuss strategies for handling cravings, stress, and weight control (C), participants strengthened their coping capacity and applied relapse-prevention techniques in daily life (O) because repetition and interactive discussion reinforced behavioral knowledge and skills, enhancing behavioral capability (M) |
|  | CMO 1.2 — Multimedia micro-lessons build practical skills | In group chats using short audio/video with in-thread Q&A (C), repetition and clarification strengthen capability (M) and support behavior change and appropriate service use (O). | [36,37,39,49,51,52,67,79,85,86,88,99-101,105,106,108,109,112,113,115]  (Randomized controlled trial, Single group/Quasi-experimental study, Non-randomized controlled trial, Mixed methods evaluation) | In post-surgery breast-cancer patients with unmet information needs, small nurse-led WeChat groups that offered multimedia education, nurse encouragement, and space for peer sharing and private chats (C) enabled timely, tailored guidance, observational learning, and low-embarrassment disclosure (M), which improved physical well-being and reduced anxiety and depression over 6 months (O) |
|  | CMO 1.3 — In-thread corrections increase accurate knowledge | When moderators correct misconceptions inside trusted group chats (C), cognitive dissonance is resolved (M) and accurate knowledge improves (O). | [39,45,71,79,101,112]  (Randomized controlled trial, Single group/Quasi-experimental study, Mixed methods evaluation) | In contexts where women aged 45‐69 in Brazil’s public health system had limited health literacy and were exposed to breast cancer misinformation through informal sources such as family and social media (C), WhatsApp-based education enabled direct questioning of myths in an informal, disinhibited environment while moderators provided evidence-based clarifications that addressed cognitive dissonance between popular beliefs and scientific facts (M), resulting in the largest knowledge gains in the “myths and truths” domain (+41 points) and improved understanding of non–risk factors for breast cancer (O). |
| Domain 2: Confidence & Motivation (IPT: Self-Efficacy, Reinforcements) | CMO 2.1 — Tailored encouragement boosts adherence | When moderators correct misconceptions inside trusted group chats (C), cognitive dissonance is resolved (M) and accurate knowledge improves (O). | [36,37,40,53,62,68,75,82,84-86,90,99,100,104,105,108,109,111]  (Randomized controlled trial, Single group/Quasi-experimental study, Pilot/ Feasibility study, Non-randomized controlled trial, Mixed methods evaluation) | In contexts where adults at risk of Type 2 diabetes participated in small, closed chat groups (10‐15 members) moderated by a health coach during the initial 3 months of a digital program (C), peer accountability, observational learning, positive reinforcement, and shared identity were activated through coach-facilitated discussion and constructive feedback (M), resulting in high engagement, mutual support, recipe and strategy sharing, goal adjustment, sustained motivation, and reported behavior change (O). |
|  | CMO 2.2 — Recognition sustains engagement | In group chats where peers/facilitators provide specific feedback and problem-solving (C), self-efficacy rises (M) and adherence is sustained (O). | [43,59,63,89,90,95,96,102,103,110,115]  (Qualitative descriptive study, Randomized controlled trial, Non-randomized controlled trial) | In contexts where older adults with digital literacy engage in structured online peer groups centered on clear behavioral goals (C), social interaction features such as photo sharing, comments, and approvals (M) foster fellowship, shared purpose, and intrinsic enjoyment, sustaining high engagement and adherence (88% retention, 87.7% adherence) and motivating continued participation beyond the formal intervention period (64% intention to continue) (O). |
|  | CMO 2.3 — Live audio/video check-ins boost follow-through | In group chats that add brief scheduled video calls or live audio check-ins (C), immediacy, non-verbal encouragement, and real-time accountability strengthen self-efficacy and motivation (M), improving adherence compared with text-only exchanges (O). | [98]  (Pilot/Feasibility study) | In weekly video-based group chats with real-time audio/video and visible nonverbal cues (C), heightened social presence and immediacy fostered cohesion and accountability (M), yielding stronger engagement and adherence signals than text-only groups (O). |
| Domain 3: Modeling & Norms (IPT: Observational Learning, Reinforcements) | CMO 3.1 — Peer strategy-sharing drives tactic uptake | When members post what they tried and with what results in group chats (C), observational learning and social comparison occur (M) and feasible tactics are adopted (O). | [37,43,44,46,47,52,53,59,61,68,69,75,77,81,82,84,85,88-90,94,95,101-104,108-111,113-116]  (Real-world program evaluation, Randomized controlled trial, Single group/ Quasi-experimental study, Qualitative descriptive study, Non-randomized controlled trial, Pilot/ Feasibility study, Mixed methods evaluation) | In contexts where school environments made medication taking stigmatized (C), participants developed workable adherence solutions (O) because they exchanged peer tips and received counselor guidance on concealment and timing (M). |
|  | CMO 3.2 — Emergent peer leaders multiply learning paths | In moderated group chats with periodic expert input that surface experienced participants (C), those members act as knowledge brokers (M) and networked learning and ongoing exchange strengthen (O). | [46,47,53,68,101]  (Pilot/Feasibility study, Randomized controlled trial, Non-randomized controlled trial, Single group/Quasi-experimental study) | In contexts where geographical and temporal barriers restricted access to professional guidance, digital group platforms enabled participants to communicate directly with experts and peers in an informal, accessible environment (C). Some participants assumed bridging roles, connecting less-engaged members to information sources, relaying and validating knowledge, and sustaining interactive discussions (M). These dynamics reduced social distance between laypeople and professionals, strengthened network cohesion, and supported ongoing peer-to-peer knowledge exchange even after professional involvement declined (O). |
| Domain 4: Safe, Supportive Environment & Access (IPT: Environment) | CMO 4.1 — Private, rule-based group chats increase participation and disclosure | In small, closed group chats with clear participation rules, explicit privacy assurances, and anonymous posting (C), participants feel safe and perceive lower social risk (M), so more people join, stay engaged, and share sensitive information (O). | [36,44,46,54,58,61,69,72,74,81,89,100,101,103,114-116]  (Real-world program evaluation, Randomized controlled trial, Single group/ Quasi-experimental study, Qualitative descriptive study, Pilot/ Feasibility study, Mixed methods evaluation) | In contexts where ALWH participated in pseudonymous WhatsApp groups moderated by a trained counselor (C), participants openly asked questions and shared experiences (O) because the platform’s privacy and peer norms created a trusted space to discuss sensitive issues (M). |
|  | CMO 4.2 — Cultural and language fit raises acceptability | Group chat messaging in participants’ primary language, using culturally familiar examples and trusted messengers (C) heightens perceived relevance and trust (M) and increases uptake of recommended actions (O). | [46,47,58,65,71,74,86,100,101,113,116]  (Pilot/ Feasibility study, Randomized controlled trial, Mixed methods evaluation, Single group/Quasi-experimental study) | In a low-literacy Yi community where pregnant women were grouped by gestational month in invite-only WeChat chats with an ANC provider and a volunteer mother (C), culturally tailored Yi-language audio/multimedia education plus in-chat Q&A improved comprehension of “what/why/when” of ANC and hospital delivery (M), yielding higher timely ANC initiation and completion and greater hospital delivery, with stronger uptake among those who joined/browsed/understood chat content (O). |
| Domain 5: Self-Regulation & Maintenance (IPT: Self-Control, Reinforcements) | CMO 5.1 — Public goals and daily check-ins drive accountability | In group chats where participants post weekly goals and provide brief daily status updates (C), public commitment and peer visibility activate accountability and self-monitoring (M), increasing day-to-day adherence and supporting maintenance (O). | [43,59,63,89,90,96,102,103,110,111,115]  (Qualitative descriptive study, Randomized controlled trial, Single group/Quasi-experimental study, Non-randomized controlled trial) | In contexts where workplace teams used WeChat to participate in a financial incentive–based walking program (C), frequent peer nudges through group messages reminding teammates to meet step goals (7‐18 per wk initially, ≥19 per wk in the medium-to-long term) (M) fostered accountability and reinforced social norms for activity, leading to sustained motivation and cumulative increases in weekly step counts, with particularly strong effects over the medium-to-long term (O). |
|  | CMO 5.2 — Scheduled nudges build automaticity | In group chats delivering regular time-based reminders (C), repeated cues strengthen cue–response links and reduce reliance on momentary motivation (M), stabilizing routine practice and sustaining behavior change (O). | [37,62,63,65-67,71,85,96,99,101,108,109,111,112]  (Randomized controlled trial, Single group/Quasi-experimental study, Pilot/ Feasibility study, Non-randomized controlled trial, Mixed methods evaluation) | In contexts where counselors shared structured, relapse-prevention reminders 3 times weekly and prompted members to discuss strategies for handling cravings, stress, and weight control (C), participants strengthened their coping capacity and applied relapse-prevention techniques in daily life (O) because repetition and interactive discussion reinforced behavioral knowledge and skills, enhancing behavioral capability (M). |
