# Supplementary material for: Using Group Chats to Drive Behavior Change in Digital Health Interventions: Scoping Review and Realist Synthesis
Source: J Med Internet Res. 2026 Apr 13;28:e88911. doi: 10.2196/88911 (PMC13075640; doi:10.2196/88911)
Supplement: Checklist 2 [file jmir-v28-e88911-s005.docx]

**PRISMA-S Checklist**

| **Section/topic** | **#** | **Checklist item** | **Location(s) Reported** |
| --- | --- | --- | --- |
| **INFORMATION SOURCES AND METHODS** | | | |
| Database name | 1 | Name each individual database searched, stating the platform for each. | Line 131-133 |
| Multi-database searching | 2 | If databases were searched simultaneously on a single platform, state the name of the platform, listing all of the databases searched. | Line 134-135 |
| Study registries | 3 | List any study registries searched. | Line 146 |
| Online resources and browsing | 4 | Describe any online or print source purposefully searched or browsed (e.g., tables of contents, print conference proceedings, web sites), and how this was done. | Line 146 |
| Citation searching | 5 | Indicate whether cited references or citing references were examined, and describe any methods used for locating cited/citing references (e.g., browsing reference lists, using a citation index, setting up email alerts for references citing included studies). | Line 147-149 |
| Contacts | 6 | Indicate whether additional studies or data were sought by contacting authors, experts, manufacturers, or others. | Line 146-147 |
| Other methods | 7 | Describe any additional information sources or search methods used. | - |
| **SEARCH STRATEGIES** | | | |
| Full search strategies | 8 | Include the search strategies for each database and information source, copied and pasted exactly as run. | See below |
| Limits and restrictions | 9 | Specify that no limits were used, or describe any limits or restrictions applied to a search (e.g., date or time period, language, study design) and provide justification for their use. | Line 140-141 |
| Search filters | 10 | Indicate whether published search filters were used (as originally designed or modified), and if so, cite the filter(s) used. | Line 141 |
| Prior work | 11 | Indicate when search strategies from other literature reviews were adapted or reused for a substantive part or all of the search, citing the previous review(s). | Line 143 |
| Updates | 12 | Report the methods used to update the search(es) (e.g., rerunning searches, email alerts). | Line 135-137 |
| Dates of searches | 13 | For each search strategy, provide the date when the last search occurred. | Line 131, Line 135 |
| **PEER REVIEW** | | | |
| Peer review | 14 | Describe any search peer review process. | Line 142-143 |
| **MANAGING RECORDS** | | | |
| Total Records | 15 | Document the total number of records identified from each database and other information sources. | Line 236-237 |
| Deduplication | 16 | Describe the processes and any software used to deduplicate records from multiple database searches and other information sources. | Line 173-175 |
|  |  |  |  |
| PRISMA-S: An Extension to the PRISMA Statement for Reporting Literature Searches in Systematic Reviews | | |  |
| Rethlefsen ML, Kirtley S, Waffenschmidt S, Ayala AP, Moher D, Page MJ, Koffel JB, PRISMA-S Group. | | |  |
| Last updated February 27, 2020. | |  |  |

Search strings

**Pubmed:** ( "group chat"[Title/Abstract] OR "group messaging"[Title/Abstract] OR "chat group"[Title/Abstract] OR "messaging group"[Title/Abstract] OR ("group"[Title/Abstract] AND (WhatsApp[Title/Abstract] OR Telegram[Title/Abstract] OR Facebook[Title/Abstract] OR WeChat[Title/Abstract] OR LINE[Title/Abstract] OR Viber[Title/Abstract] OR Signal[Title/Abstract] OR "messaging app*"[Title/Abstract] OR "messenger app*"[Title/Abstract] OR "instant messaging"[Title/Abstract] OR "social messaging"[Title/Abstract]) ) ) AND ( "health promotion"[Title/Abstract] OR "health intervention*"[Title/Abstract] OR "health education"[Title/Abstract] OR "health communication"[Title/Abstract] OR "behavior change"[Title/Abstract] OR "health awareness"[Title/Abstract] OR "health attitudes"[Title/Abstract] OR "health literacy"[Title/Abstract] OR "disease prevention"[Title/Abstract] OR "disease screening"[Title/Abstract] OR "early detection"[Title/Abstract] OR "preventive health"[Title/Abstract] OR "health screening"[Title/Abstract] OR "public health"[Title/Abstract] OR "self-management"[Title/Abstract] OR "patient support"[Title/Abstract] OR "peer support"[Title/Abstract] ) AND ( intervention*[Title/Abstract] OR trial[Title/Abstract] OR RCT[Title/Abstract] OR "randomized controlled trial"[Title/Abstract] OR "controlled trial"[Title/Abstract] OR "clinical trial"[Title/Abstract] OR "experimental study"[Title/Abstract] OR "program evaluation"[Title/Abstract] OR "evaluation study"[Title/Abstract] OR "pilot study"[Title/Abstract] OR "feasibility study"[Title/Abstract] OR "quasi-experimental"[Title/Abstract] OR "pre-post"[Title/Abstract] ) AND ("2005/01/01"[Date - Publication] : "3000"[Date - Publication]) AND english[Language] NOT ( review[Publication Type] OR "systematic review"[Title] OR "scoping review"[Title] OR "narrative review"[Title] OR "meta-analysis"[Publication Type] )

**Embase / MEDLINE (via Ovid):** ( 'group chat':ti,ab OR 'group messaging':ti,ab OR 'chat group':ti,ab OR 'messaging group':ti,ab OR ( (group NEAR/10 (whatsapp OR telegram OR facebook OR wechat OR line OR viber OR signal OR 'instant messaging' OR 'messenger app' OR 'messaging app' OR 'social messaging' OR 'social media')):ti,ab ) ) AND ( 'health promotion'/exp OR 'health education'/exp OR 'health communication'/exp OR 'health behavior'/exp OR 'self management'/exp OR 'chronic disease prevention'/exp OR 'screening'/exp OR 'public health'/exp OR 'peer support'/exp ) AND ( 'randomized controlled trial'/exp OR 'clinical trial'/exp OR 'pilot study'/exp OR 'feasibility study'/exp OR 'quasi experimental study'/exp OR 'program evaluation'/exp OR 'intervention study'/exp ) AND [english]/lim AND [2005-2025]/py NOT ('systematic review'/exp OR 'scoping review':ti OR 'narrative review':ti OR 'meta analysis'/exp)

**Web of Science:** TS=( "group chat" OR "group messaging" OR "chat group" OR "messaging group" OR "whatsapp group" OR "telegram group" OR "facebook group" OR "wechat group" OR "line group" OR "viber group" OR "signal group" OR (group NEAR/10 (whatsapp OR telegram OR facebook OR wechat OR line OR viber OR signal OR "instant messaging" OR "messenger app" OR "messaging app" OR "social messaging")) ) AND TS=( "health promotion" OR "health intervention*" OR "health education" OR "health communication" OR "behavior change" OR "health awareness" OR "health literacy" OR "disease prevention" OR "disease screening" OR "self-management" OR "treatment adherence" OR "peer support" ) AND TS=( intervention* OR trial OR RCT OR "randomized controlled trial" OR "controlled trial" OR "clinical trial" OR "experimental study" OR "program evaluation" OR "evaluation study" OR "pilot study" OR "feasibility study" OR "quasi-experimental" OR "pre-post" )

**Scopus:** TITLE-ABS-KEY( "group chat" OR "group messaging" OR "chat group" OR "messaging group" OR "whatsapp group" OR "telegram group" OR "facebook group" OR "wechat group" OR "line group" OR "viber group" OR "signal group" OR (group W/10 (whatsapp OR telegram OR facebook OR wechat OR line OR viber OR signal OR "instant messaging" OR "messenger app" OR "messaging app" OR "social messaging" OR "social media")) ) AND TITLE-ABS-KEY( "health promotion" OR "health intervention*" OR "health education" OR "health communication" OR "behavior change" OR "health awareness" OR "health attitudes" OR "health literacy" OR "disease prevention" OR "disease screening" OR "early detection" OR "preventive health" OR "health screening" OR "public health" OR "self-management" OR "treatment adherence" OR "patient support" OR "peer support" ) AND TITLE-ABS-KEY( intervention* OR trial OR RCT OR "randomized controlled trial" OR "controlled trial" OR "clinical trial" OR "experimental study" OR "program evaluation" OR "evaluation study" OR "pilot study" OR "feasibility study" OR "quasi-experimental" OR "pre-post" )
